# Assessment of Acute Appendicitis in Pregnant Women: A Systematic Review of Current Evidence

**DOI:** 10.7759/cureus.107410

**Published:** 2026-04-20

**Authors:** Nuaman A Danawar, Mohammad Mahmood Almohamad, Khaldon A Alkhoms, Firas Mohmad Alahmadatea, Rasoul M Alyasin, Mahdi Abdulrasoul, Khalid Mansour Alammar, Bader AlQusaibi

**Affiliations:** 1 General Surgery, Security Forces Hospital, Dammam, SAU; 2 General Surgery, Aster Sanad Hospital, Riyadh, SAU; 3 Surgery, Security Forces Hospital, Dammam, SAU; 4 General Surgery, National Guard Hospital, Al-Madinah, SAU; 5 General Surgery, Qatif Central Hospital, Al Qatif, SAU; 6 Medicine, King Abdulaziz University, Jeddah, SAU

**Keywords:** acute appendicitis, clinical scoring systems, diagnosis, diagnostic accuracy, magnetic resonance imaging (mri), pregnancy

## Abstract

The most frequent nonobstetric surgical emergency during pregnancy is acute appendicitis (AA); however, diagnosing it is still difficult because of anatomical and physiological changes. While magnetic resonance imaging (MRI) has emerged as a possible substitute, traditional methods such as clinical ratings and ultrasound have drawbacks. The latest research on the diagnostic accuracy of clinical scores, laboratory indices, and MRI for AA in pregnant women is compiled in this systematic review. Preferred Reporting Items for Systematic Reviews and Meta-Analyses standards were followed in conducting a systematic review. PubMed, Embase, Cochrane, Scopus, and Web of Science databases were searched from January 2020 to December 2025 for studies evaluating diagnostic tools in pregnant women with suspected appendicitis. Using the Quality Assessment of Diagnostic Accuracy Studies-2 tool, two reviewers independently searched for studies, retrieved data, and evaluated risk of bias. Due to methodological variability, a narrative synthesis was conducted. Fifteen studies were included. MRI demonstrated consistently high specificity and negative predictive value, significantly reducing negative appendectomy rates when used after an inconclusive ultrasound. Clinical scoring systems (e.g., Alvarado, Raja Isteri Pengiran Anak Saleha appendicitis, and Appendicitis Inflammatory Response) showed poor positive predictive value and limited reliability for confirming appendicitis, though they retained utility for initial triage. Compared with conventional indicators such as white blood cell count, novel composite laboratory indices, including the Systemic Immune-Inflammation Index (SII) and Pregnancy Index, showed promising diagnostic accuracy and high specificity. MRI is the cornerstone of noninvasive diagnosis for suspected appendicitis in pregnancy, offering high accuracy and safety. Clinical scores and basic laboratory markers are insufficient for definitive diagnosis but may aid in initial assessment. A sequential diagnostic algorithm utilizing ultrasound first, followed by MRI if inconclusive, is supported by current evidence to optimize maternal and fetal outcomes. Future research should focus on validating pregnancy-specific diagnostic indices and protocols in prospective multicenter studies.

## Introduction and background

With an estimated frequency of one in 500 to one in 2,000 pregnancies, acute appendicitis (AA) is the most common nonobstetric surgical emergency that occurs during pregnancy [1,2]. On the other hand, a needless surgical procedure exposes the expectant mother and her unborn child to the hazards associated with anesthesia and surgery. As a result, diagnosing right lower quadrant discomfort during pregnancy requires striking a compromise between sensitivity and specificity, which presents special difficulties.

The typical physiological and anatomical changes that characterize pregnancy greatly complicate the diagnostic process. The appendix is moved from its usual placement in the right lower quadrant as the pregnancy goes on by the growing uterus, which usually rotates it laterally and superiorly [3]. In contrast to the classic presentation of appendicitis, this anatomical shift frequently causes pain to manifest in the right flank or right upper quadrant. Additionally, the symptoms of an acute abdominal process might be confused with the physiological leukocytosis, nausea, vomiting, and anorexia that are frequently observed during pregnancy [4]. Due to these characteristics, pregnant populations have historically had a greater risk of negative appendectomy, up to 20%-35% in certain series, compared to around 10%-15% in the general population [5].

Traditional diagnostic modalities are of limited utility in this context. Clinical scoring systems, such as the Appendicitis Inflammatory Response (AIR) and Alvarado scoring, which are validated in the general adult population, perform suboptimally in pregnancy due to their reliance on symptoms and laboratory values that are altered by the gravid state [6]. Despite being safe and radiation-free, graded-compression ultrasonography (US) sometimes yields nondiagnostic results, especially in the second and third trimesters, due to technical challenges posed by the larger uterus and the appendix's changing location [7]. Computed tomography (CT), although highly accurate, involves ionizing radiation and is often avoided during pregnancy because of the possible teratogenic dangers [8]. This diagnostic conundrum necessitates the use of highly accurate, noninvasive imaging alternatives.

Magnetic resonance imaging (MRI) has become a vital diagnostic technique in recent years for assessing suspected appendicitis during pregnancy. Its superior contrast between soft tissues, absence of ionizing radiation, and high reported sensitivity and specificity make it an ideal modality. Concurrently, research continues to refine laboratory biomarkers and develop pregnancy-specific clinical algorithms to improve diagnostic precision. However, the evidence surrounding the optimal application, comparative accuracy, and integration of these various tools, ranging from advanced MRI protocols and novel inflammatory indices to modified scoring systems, remains dispersed and has not been recently synthesized comprehensively. Thus, the purpose of this systematic review is to evaluate and compile the available data about the diagnostic precision of laboratory indices, clinical grading systems, and MRI for AA in pregnant women.

## Review

Methodology

To guarantee scientific rigor and transparent reporting, the systematic review was carried out in compliance with the Preferred Reporting Items for Systematic Reviews and Meta-Analyses (PRISMA) criteria [9].

Search Strategy and Information Sources

To find all pertinent published research, a thorough and methodical literature search was conducted. PubMed/MEDLINE, Embase (via Ovid), the Cochrane Library, Scopus, and the Web of Science Core Collection are the five main electronic bibliographic databases that were searched. An expert medical librarian created the search method, which combined a number of restricted vocabulary phrases (e.g., Medical Subject Headings terms in PubMed such as "Appendicitis", "Pregnancy", "Diagnostic Imaging", and "Magnetic Resonance Imaging"). It includes free-text terms associated with the ailment, population, and diagnostic techniques. To capture the most recent data and developments in diagnostic imaging and biomarkers, the search was limited to studies published in English over a five-year period, from January 2020 to December 2025. To optimize sensitivity, the research design was initially unrestricted. To guarantee repeatability, an appendix contains the whole search technique for at least one database (PubMed).

Study Selection Process

The web-based systematic review program, Rayyan (Rayyan Systems Inc., Cambridge, MA), was used to carry out the research selection procedure in two stages [10]. During the initial stage, duplicate citations were eliminated both automatically and manually after all records obtained from the database searches were loaded into Rayyan. The titles and abstracts of each unique record were compared with the predetermined eligibility criteria by two separate reviewers. Research was included if it involved pregnant women with suspected AA, evaluated one or more diagnostic tools such as clinical scoring systems, laboratory indices, ultrasound, or MRI protocols, and reported quantitative diagnostic accuracy outcomes or provided sufficient data to calculate them. At this point, studies that were not published in English, case reports, narrative reviews, editorials, and animal studies were all disregarded. Full texts of all potentially eligible papers were obtained and evaluated separately by the same two reviewers for ultimate inclusion in the second step. To reach consensus, any differences or conflicts regarding research eligibility at each stage were discussed or, if required, consulted with a third senior reviewer. This stringent, two-reviewer procedure guaranteed the validity and impartiality of the research selection [11].

Data Extraction and Management

To guarantee uniform and precise data gathering from every included study, a standardized, pilot-tested data extraction form was created in Microsoft Excel (Microsoft Corporation, Redmond, WA). Two reviewers separately extracted the data, which was then cross-checked. The source publication was reexamined to reconcile any inconsistencies, and the extracted data encompassed several key domains: study characteristics, including the first author, publication year, journal, study location, study design (such as retrospective cohort or case-control), and study period; participant characteristics, which included sample size, mean or median age, gestational age distribution, and clinical presentation; diagnostic test details, providing a comprehensive description of the index test(s) evaluated (such as specific MRI sequences, formulas for laboratory indices, and components of clinical scores), along with any specified cutoff values; reference standard, which referred to the method used to confirm the final diagnosis (including histopathological examination after appendectomy and clinical and imaging follow-up); and outcomes, detailing the primary diagnostic accuracy measures reported in the studies, which encompassed diagnostic accuracy, area under the receiver operating characteristic curve (area under the curve (AUC)), positive predictive value (PPV), negative predictive value (NPV), sensitivity, and specificity. The values were computed manually when these measures were not specified explicitly, but raw data were available.

Assessment of Risk of Bias in Included Studies

The Quality Assessment of Diagnostic Accuracy Studies-2 (QUADAS-2) tool was used to objectively evaluate the methodological quality and risk of bias in each included diagnostic accuracy study [12]. Patient Selection, Index Test, Reference Standard, and Flow and Timing are the four primary areas in which the QUADAS-2 instrument evaluates bias. The danger of bias in each category is assessed as "high", "low", or "unclear", depending on signaling questions specific to the review's setting. Certain standards were set for this review. For instance, a case-control design with healthy controls was noted for possible bias in the "Patient Selection" area, but a sequential or random sample of pregnant women with probable appendicitis was deemed low risk. The "Index Test" domain required interpretation without knowledge of the reference standard result (blinding). A satisfactory, conclusive diagnosis (histopathology or rigorous follow-up) must be used for the "Reference Standard" domain. Each study was subjected to independent application of the QUADAS-2 criteria by two reviewers; differences were settled by consensus.

Data Synthesis and Analysis

A formal quantitative meta-analysis to generate pooled summary estimates of sensitivity and specificity was considered unsuitable due to the expected and observed clinical and methodological heterogeneity among the included studies, differences in study design, index test protocols, laboratory cutoff values, and patient populations. Rather, an organized tabulation of the retrieved data was used to assist a narrative synthesis technique. The findings are organized thematically based on the type of diagnostic tool evaluated, which includes advanced imaging techniques such as MRI and its various sequences or scales, clinical scoring systems, and laboratory-based biomarkers and indices. Within each theme, study results are described, compared, and contrasted, highlighting patterns of diagnostic performance, such as the consistently high NPV for MRI, sources of variation, and the clinical applicability of the findings. This synthesis offers a thorough and cohesive overview of the available data on the diagnosis of AA in expectant mothers.

Results

**Figure 1:**
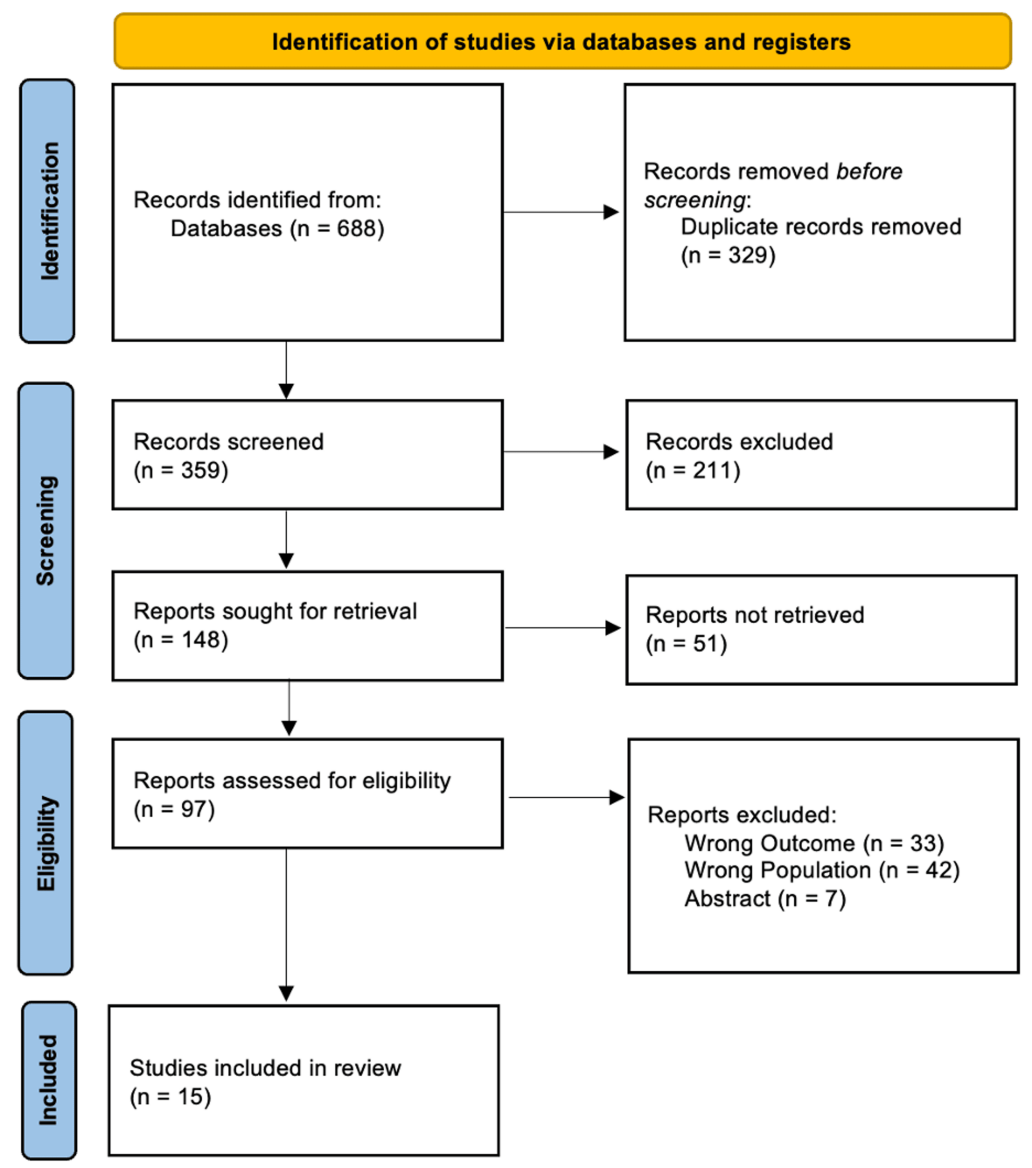
PRISMA flow diagram of study selection PRISMA: Preferred Reporting Items for Systematic Reviews and Meta-Analyses Source: [9]

Figure 1 shows the methodical study selection procedure, as shown in the PRISMA flow diagram [9]. A total of 688 documents were found in the initial database searches; 211 entries were excluded after 359 records passed title and abstract screening following the elimination of 329 duplicates. Of the 148 reports for which full-text retrieval was attempted, 51 were unavailable, leaving 97 for a thorough eligibility evaluation. Of these, 82 were excluded for the following reasons: 33 did not report relevant diagnostic outcomes, 42 did not focus on the correct patient population (pregnant women with suspected appendicitis), and seven were conference abstracts without accessible full data. Consequently, 15 studies met all criteria and were included in the final systematic review.

As shown in Table 1, which illustrates the demographic and methodological characteristics of the included studies [13-27], the 15 papers that made up this systematic review provide substantial information on the difficulty of diagnosing AA in pregnant women. Geographically, the research is predominantly concentrated in Turkey, with eight studies [14,16,18,19,21-24], and additional significant work from Israel [15,20], Taiwan [13], Vietnam [25,27], Lithuania [17], and other regions, providing a multinational perspective. Methodologically, the landscape is overwhelmingly defined by retrospective designs, including diagnostic cohorts, case-control studies, and comparative analyses. Sample sizes exhibit considerable variation, ranging from smaller cohorts of 25-42 patients [23,25] to more substantial studies encompassing 76-180 participants [14,15,17,20]. This heterogeneity in design and scale is a critical factor for consideration when synthesizing the evidence. The study populations are consistently well-defined, focusing exclusively on pregnant women presenting with suspected AA, with the majority utilizing histopathological confirmation from appendectomy as the definitive reference standard, thereby grounding the diagnostic accuracy data in objective outcomes.

**Table 1:** Demographic and methodological characteristics of included studies AA: acute appendicitis; MRI: magnetic resonance imaging; NM: not mentioned; HPW: healthy pregnant woman; UPW: unhealthy pregnant woman; HW: healthy woman; UW: unhealthy woman; US: ultrasound

Study	Study location	Study design	Sample size	Study population	Mean age (years)	Gestational age	Additional notes
Wong et al. [13]	Taiwan	Retrospective diagnostic cohort	80 patients	Pregnant women with suspected AA who underwent MRI	NM	NM	Study period: January 2018-December 2020
Altuğ et al. [14]	Turkey	Retrospective, single-center, case-control	76 pregnant women (38 cases, 38 controls)	Pregnant women with histopathologically confirmed AA and healthy pregnant controls	NM	≥12 weeks	Cases diagnosed via pathology report
Bekhor et al. [15]	Israel	Retrospective cohort	180 pregnant women (28 AA, 152 controls)	Pregnant women who underwent MRI for suspected AA	NM	NM	Used national database for follow-up; period: 2013-2023
Sezİklİ et al. [16]	Turkey	Retrospective, case-control	120 patients (4 groups of 30)	Groups: healthy pregnant women (HPW), pregnant women with AA (UPW), healthy nonpregnant women (HW), nonpregnant women with AA (UW)	NM	NM	Study period: 2015-2021
Lukenaite et al. [17]	Lithuania	Retrospective, single-center comparative	76 pregnant women (38 in US-only, 38 in US+MRI group)	Pregnant women admitted with suspected AA	NM	NM	Study period: January 2012-December 2019
Çomçalı et al. [18]	Turkey	Retrospective diagnostic	140 female patients (35 pregnant with AA, 105 nonpregnant with AA)	Women who had an appendectomy for suspected AA, both pregnant and not	Age range: 18-49 years	NM	Selected from 1,542 screened patients
Bardakçi et al. [19]	Turkey	Retrospective diagnostic	53 pregnant women	Pregnant women who underwent surgery for suspected AA	28.58 (range: 18-44)	1st, 2nd, and 3rd trimesters	Study period: February 2014-December 2018
Bufman et al. [20]	Israel	Retrospective, single-center	167 pregnant women	Pregnant women who underwent emergent MRI for suspected AA	NM	NM	Study period: April 2013-June 2021
Akbas et al. [21]	Turkey	Retrospective with prospective control group	96 pregnant women (32 AA, 32 under observation, 32 healthy)	Three groups: pregnant with AA (surgically confirmed), pregnant under observation for abdominal pain, healthy pregnant controls	29.20 ± 4.47	NM	Study period: September 2015-December 2019
Mantoglu et al. [22]	Turkey	Retrospective diagnostic comparison	158 women (79 pregnant, 79 nonpregnant)	Women who had an appendectomy for suspected AA, both pregnant and not	NM	NM	Study period: May 2014-May 2019
Akın et al. [23]	Turkey	Retrospective case series	42 pregnant women	Pregnant women who underwent appendectomy	30 ± 6	All trimesters	NM
Somuncu et al. [24]	Turkey	Retrospective, case-control	117 women (39 pregnant with AA, 39 nonpregnant with AA, 39 healthy pregnant)	Three groups: pregnant with AA, nonpregnant with AA, healthy pregnant controls	NM	NM	NM
Hung et al. [25]	Vietnam	Retrospective diagnostic comparison	25 pregnant patients	Pregnant women with suspected AA who underwent 1.5T MRI	NM	NM	NM
Erdoğan et al. [26]	Turkey	Retrospective, case-control	1,229 women (58 pregnant, 1,171 nonpregnant)	Women who had appendectomies, both pregnant and not pregnant	The pregnant group was significantly younger (p < 0.0001, d = -0.532)	NM	Study period: January 2010-January 2021
Thanh Thi Nguyen et al. [27]	Vietnam (University Medical Center, Ho Chi Minh City)	Retrospective diagnostic	179 pregnant women	Pregnant women with suspected AA who underwent MRI	29.7 ± 4.8 (range: 18-46)	NM	Study period: January 2016-October 2023

Table 1 further elucidates the specific diagnostic tools under investigation, which form the core of this review. The studies can be thematically categorized into three primary groups: advanced imaging techniques, novel laboratory indices, and clinical scoring systems. The imaging research, exemplified by Wong et al. [13], Hung et al. [25], and Thanh Thi Nguyen et al. [27], delves into optimizing MRI protocols, assessing specific sequences like diffusion-weighted imaging and three-dimensional T1-weighted gradient-echo, and developing quantitative scoring scales to objectify interpretation. Laboratory-based studies, such as those by Altuğ et al. [14] and Sezİklİ et al. [16], investigate the diagnostic value of both new composite indices like the Pregnancy Index (PGIndex) and the Systemic Immune-Inflammation Index (SII) as well as well-known inflammatory markers like white blood cell (WBC) and neutrophil-to-lymphocyte ratio (NLR). Bekhor et al. represented the third group [15]. Mantoglu et al. [22] and Bardakçi et al. [19] conducted critical comparisons of various clinical scoring systems (e.g., Alvarado, Raja Isteri Pengiran Anak Saleha appendicitis (RIPASA), and AIR) against each other and against imaging results, aiming to identify the most reliable triage tool for this unique patient population.

The diagnostic performance metrics extracted from these studies, summarized in Table 2, reveal a complex and nuanced picture of the current evidence. MRI consistently shows good specificity and NPV for advanced imaging. For example, the MRI appendicitis scale has a 90.2% specificity and a 96.6% sensitivity [13], while MRI as a modality was reported to have an NPV of 96% and a specificity of 100% in different studies [15,17]. This high NPV is clinically paramount, as it effectively rules out AA and prevents unnecessary surgery. Among laboratory markers, the novel composite indices show particular promise; the SII achieved an AUC of 0.790 for AA and 0.812 for complicated AA [14], and the PGIndex demonstrated a high specificity of 96.7% [16]. In contrast, the performance of routine hematological parameters such as WBC and NLR was more variable and generally moderate, as observed in studies by Somuncu et al. [24] and Akbas et al. [21].

**Table 2:** Diagnostic findings and outcomes of included studies MRI: magnetic resonance imaging; DWI: diffusion-weighted imaging; PPV: positive predictive value; SII: systemic immune-inflammation index; AA: acute appendicitis; AUC: area under the curve; RIPASA: Raja Isteri Pengiran Anak Saleha Appendicitis Score; AIR: Appendicitis Inflammatory Response; MRI: magnetic resonance imaging; NPV: negative predictive value; PGIndex: Pregnancy index; WBC: white blood cell; CRP: C-reactive protein; NLR: neutrophil-to-lymphocyte ratio; iMA: ischemia-modified albumin; PLR: platelet-to-lymphocyte ratio; US: ultrasound; DNI: delta neutrophil index; AS: Alvarado Score; GI: gastrointestinal; CAR: C-reactive protein-to-albumin ratio, and LCR: lymphocyte-to-C-reactive protein ratio; MPV: mean platelet volume; RDW: red cell distribution width; PLT: platelet; ALC: absolute lymphocyte count; T2WI: T2-weighted imaging

Study	Diagnostic tool/index evaluated	Reference standard	Key diagnostic performance metrics	Main conclusion/findings
Wong et al. [13]	MRI appendicitis scale (+DWI)	Pathological or clinical follow-up (implied)	Sens: 96.6%, Spec: 90.2%, PPV: 84.8%. Odds ratio: 22.3 per 1-point increase in scale	An important independent predictor is the MRI appendicitis scale. The diagnostic value is increased by adding DWI
Altuğ et al. [14]	SII	Histopathology	For AA: Sens 82.0%, Spec 66.7%, cutoff: 840.13, AUC: 0.790. For complicated AA: Sens 66.7%, Spec 91.3%, cutoff: 2,301.66, AUC: 0.812.	SII is a cost-effective and powerful marker for diagnosing both simple and complicated AA in pregnancy
Bekhor et al. [15]	Clinical scores (Alvarado, Tzanakis, RIPASA, AIR) and MRI	Pathology (AA group) and follow-up (control group)	PPV of scores: Alvarado 38%, Tzanakis 57%, RIPASA 22%, AIR 12%. NPV of Scores: 80%-86%. MRI: PPV 62%, NPV 96%	Most AA patients had negative clinical scores. MRI had a very high NPV. Discrepancy highlights diagnostic complexity
Sezİklİ et al. [16]	PGIndex: WBC, CRP, NLR, iMA, PLR)	Final diagnosis (pathology/clinical)	Cutoff >10.62: Sens 73.3%, Spec 96.7%, PPV 95.7%, NPV 78.7%, accuracy 85%	One important indication with a high specificity for identifying AA in pregnant women is the new PGIndex
Lukenaite et al. [17]	MRI after an inconclusive ultrasound	Surgery/pathology or clinical follow-up	MRI performance: Sens 83.3%, Spec 100%. Unnecessary surgery: 2.6% (US+MRI) vs. 26.3% (US-only), p = 0.007	Using MRI after an inconclusive US significantly reduces unnecessary surgery rates and shortens hospital stay
Çomçalı et al. [18]	Tzanakis Score modified with DNI	Histopathology	Tzanakis: Sens 84.85%, accuracy 85.71%. Tzanakis+DNI: Sens 93.94%, accuracy 94.29%, NPV 50%	Tzanakis is effective. Modifying it with DNI improves sensitivity and accuracy for diagnosing AA in pregnancy
Bardakçi et al. [19]	AS vs. AIR Score	Histopathology	In the 1st/2nd trimester, most patients had AS ≥7, while fewer had AIR ≥9. In the 3rd trimester, both scores were less predictive	There is no clear advantage between AS and AIR when it comes to detecting AA in pregnant women
Bufman et al. [20]	Emergent MRI	Final clinical/imaging diagnosis	AA on MRI: 20.9% (35/167). Alternative diagnosis on MRI: 18% (30/167): gynecological (56.7%), urological (26.7%), GI (20%)	MRI is valuable not only for diagnosing AA but also for identifying alternative causes of abdominal pain in pregnancy
Akbas et al. [21]	Inflammatory markers (WBC, NLR, CAR, and LCR)	Pathology (AA group)/resolution without surgery (observation group)	WBC, CAR, and NLR were significantly higher; LCR was significantly lower in the AA group (p = 0.001). All were independent predictors in logistic regression	WBC, NLR, CAR, and LCR can assist clinicians in diagnosing AA when combined with history and examination
Mantoglu et al. [22]	Nine different clinical scoring systems	Histopathology	RIPASA score in pregnant women: PPV 94.40%, NPV 44%, Sens 78.46%, Spec 78.57%	A pregnancy-specific system is still required, although the RIPASA score outperformed the other nine systems for expectant mothers
Akın et al. [23]	Routine laboratory parameters (neutrophil, WBC, NLR, PLR, etc.)	Histopathology	No significant difference in lab parameters between the normal appendix and AA groups (p > 0.05). Negative appendectomy rate higher in pregnant vs. controls (p = 0.001)	For diagnosis, laboratory values are not enough. To lower the rate of negative appendectomy, MRI should be taken into consideration
Somuncu et al. [24]	Hemogram parameters (WBC, neutrophil, NLR, PLR, MPV, RDW)	Histopathology or health status	NLR: AUC 0.667, cutoff 9.23 (Sens 46.2%, Spec 92.3%). WBC: cutoff 14,155 (Sens 51.3%, Spec 82.1%). PLR: cutoff 157.6 (Sens 51.3%, Spec 82.1%)	Neutrophil count, WBC, and PLR were the most valuable inflammatory parameters. NLR had the largest AUC
Hung et al. [25]	MRI sequences: 3D T1W-GRE vs. 2D T1W in/out phase GRE	Definitive diagnosis (method not specified)	Group B (T2WI + 3D T1W-GRE): Sens 80%, Spec 100%, PPV 100%, NPV 95.2%, Accuracy 96%. Group A (T2WI only): Accuracy 88%	3D T1W-GRE sequences improve diagnostic accuracy compared to T2WI alone. Adding in/out phase sequences did not increase accuracy
Erdoğan et al. [26]	Complete blood count parameters (PLT, MPV, ALC)	Histopathology	MPV was the only independent predictor in pregnant women (OR: 1.952). MPV cutoff 9.6 fL: Sens 63.5%, Spec 67.7%, PPV 94.2%, NPV 17.3%. MPV cutoff 10 fL: Sens 55.8%, Spec 100%, PPV 100%	MPV might be a useful biomarker for diagnosing AA in pregnant women, potentially reducing negative laparotomy rates
Thanh Thi Nguyen et al. [27]	MRI pulse sequences (T2W, T1W, DWI)	Surgical and histopathological findings	T2W alone: Sens 93.5%, Spec 92.3%. T2W+T1W+DWI: Sens 96.8%, Spec 94.9%. T1 bright appendix sign: NPV 95.6%	T2W is highly useful. Adding T1W and DWI increases sensitivity and specificity, boosting diagnostic confidence

The evaluation of clinical scoring systems, as shown in Table 2 [15,18,19,22], indicates a significant diagnostic challenge. While some scores, like the Tzanakis and RIPASA, showed utility, with the latter having a PPV of 94.40% in one study [22], their overall predictive value in pregnancy appears limited. The comparative analysis by Bekhor et al. [15] starkly illustrated this, revealing low PPVs (12%-57%) for all major scoring systems, despite high NPVs. This underscores a critical finding across the dataset: while clinical scores and basic labs are useful for ruling out disease, their ability to confirm AA is insufficient. Consequently, the evidence strongly supports the pivotal role of MRI as a follow-up investigation after an inconclusive ultrasound, a strategy proven by Lukenaite et al. [17] to significantly reduce negative appendectomy rates from 26.3% to 2.6%. The collective data argue for a sequential diagnostic algorithm that leverages clinical assessment for initial triage but relies on advanced imaging for definitive preoperative diagnosis.

Table 3 presents the risk-of-bias assessment using the QUADAS-2 tool [12]. All studies show a consistently high overall risk of bias due to flawed patient selection, despite having low-risk index tests, reference standards, and flow/timing.

**Table 3:** Risk of bias assessment using the QUADAS-2 tool QUADAS-2: Quality Assessment of Diagnostic Accuracy Studies-2 Source: [12]

Study	Patient selection	Index test	Reference standard	Flow and timing	Overall risk
Wong et al. [13]	High	Low	Low	Low	High
Altuğ et al. [14]	High	Low	Low	Low	High
Bekhor et al. [15]	High	Low	Low	Low	High
Sezİklİ et al. [16]	High	Low	Low	Low	High
Lukenaite et al. [17]	High	Low	Low	Low	High
Çomçalı et al. [18]	High	Low	Low	Low	High
Bardakçi et al. [19]	High	Low	Low	Low	High
Bufman et al. [20]	High	Low	Low	Low	High
Akbas et al. [21]	High	Low	Low	Low	High
Mantoglu et al. [22]	High	Low	Low	Low	High
Akın et al. [23]	High	Low	Low	Low	High
Somuncu et al. [24]	High	Low	Low	Low	High
Hung et al. [25]	High	Low	Low	Low	High
Erdoğan et al. [26]	High	Low	Low	Low	High
Thanh Thi Nguyen et al. [27]	High	Low	Low	Low	High

Discussion

The findings confirm that MRI has cemented its role as the cornerstone of noninvasive diagnostic evaluation following an inconclusive ultrasound. The consistently high specificity (90.2%-100%) and NPV (95.2%-96%) reported across multiple studies [13,15,17,25,27] are its most critical attributes. This performance translates directly into a profound clinical benefit: the significant reduction of negative appendectomy rates. Our data, particularly from Lukenaite et al. [17], demonstrate that integrating MRI into the diagnostic algorithm can lower unnecessary surgery rates from over 26% to under 3%. This aligns with and strengthens the conclusions of earlier pivotal studies. For instance, a landmark prospective study by Akbaş established MRI as highly accurate for suspected abdominal aortic aneurysm in pregnancy, demonstrating 100% sensitivity and 94% specificity in a group of 114 patients [28]. Similarly, a meta-analysis by Leeuwenburgh et al., which included data from 14 studies, found that MRI had a pooled sensitivity of 94% and specificity of 97% for identifying AA in pregnant women, effectively minimizing fetal radiation exposure from CT scans without compromising diagnostic confidence [29]. Our review reinforces these conclusions with newer data, including the validation of quantitative MRI scales that further objectify interpretation [13]. The evolution from simply using MRI to developing structured reporting scales, as shown by Wong et al. [13], represents a significant advancement toward standardizing imaging assessment and reducing interobserver variability, a known limitation in earlier applications.

In stark contrast to MRI's reliable performance, our synthesis reveals the substantial limitations of clinical scoring systems when applied to pregnant populations. While instruments such as the AIR scores, RIPASA, and Alvarado are validated and useful in the broader adult population [30], their adaptation to pregnancy is problematic. The physiological changes of pregnancy, such as leukocytosis, nausea, and shifting abdominal pain, directly confound key components of these scores. The PPV for these systems was consistently low in our included studies, ranging from 12% for the AIR score to 57% for the Tzanakis score [15]. Notably, Bekhor et al. [15] found that a majority of pregnant women with confirmed AA presented with negative clinical scores. This finding is corroborated by prior research; a study specifically evaluating the Alvarado score in pregnancy found it had a sensitivity of only 62% and a negative appendectomy rate of 33% when used as the primary decision tool [31]. Our review, particularly the work of Mantoglu et al. [22], suggests that the RIPASA score may have a relative advantage in this cohort, but its PPV of 94.4% in their study requires external validation and may not translate to all settings. The collective evidence strongly argues against relying on these generic scoring systems for definitive diagnosis in pregnancy. Their utility likely remains in initial triage, where a high score can increase suspicion, but a low or intermediate score cannot reliably exclude the disease, necessitating further imaging.

The search for an accurate, rapid, and inexpensive laboratory biomarker for AA in pregnancy remains an active and crucial area of research, as reflected in several of our included studies. Because of gestational physiologic leukocytosis and the unpredictable acute-phase response during pregnancy, traditional indicators such as WBC count and C-reactive protein (CRP) are infamously inaccurate [32]. Our data confirm this, showing only moderate sensitivity and specificity for WBC and differential counts [21,23,24]. Consequently, recent research has pivoted toward investigating novel composite indices that integrate multiple parameters to better reflect the inflammatory state. Our review highlights promising candidates in this domain. Platelet, neutrophil, and lymphocyte counts are included in the SII, which demonstrated a strong AUC of 0.790 for AA and 0.812 for complex AA [14]. Similarly, the novel PGIndex, combining WBC, CRP, NLR, platelet-to-lymphocyte ratio (PLR), and ischemia-modified albumin, demonstrated excellent specificity (96.7%) [16]. These indices expand on earlier research examining ratios such as the PLR and NLR. Markar et al.'s meta-analysis concluded that NLR had moderate diagnostic accuracy for AA in general populations but noted significant heterogeneity and called for population-specific cutoffs [32]. The research conducted by Altuğ et al. [14] and Sezİklİ et al. [16] answers this call by developing and validating indices specifically in pregnant cohorts. Another innovative approach, modifying established clinical scores with novel hematological parameters, was explored by Çomçalı et al. [18], who enhanced the Tzanakis score by incorporating the Delta Neutrophil Index, improving its sensitivity. These efforts mirror the trajectory of research in nonpregnant populations, such as the creation of the AIR score, which performed better by integrating inflammatory response criteria than the Alvarado score [33]. The translation and adaptation of such multimodal thinking to pregnancy is a logical and necessary step forward.

Based on the synthesized evidence, we propose a refined, stepwise diagnostic algorithm for suspected AA in pregnancy. The initial assessment must include a detailed history, a physical examination that recognizes the anatomical shifts of pregnancy, and basic laboratory studies. A generic clinical score (e.g., Alvarado and RIPASA) can be calculated for context, but should not be definitive. Graded-compression US should be used as the first imaging modality because it is safe and easily accessible, and can make a definitive diagnosis if a normal appendix is seen in the right lower quadrant or if a noncompressible, dilated (>6 mm) appendix with periappendiceal fluid is clearly visible [34,35]. However, as supported by our data and previous literature, US often yields nondiagnostic results in pregnancy, particularly in later gestation, with visualization rates as low as 16% in some series [36]. In cases of inconclusive or negative US with persistent clinical suspicion, MRI without contrast should be performed without delay. Our review and numerous guidelines now position MRI not as a problem-solving tool but as a necessary second-line investigation [7,37]. The high NPV of MRI is its greatest strength, allowing for safe discharge and avoidance of surgery when negative. If MRI is positive for AA, surgical management (preferably laparoscopic, especially in the first and second trimesters) should proceed [38]. CT should be reserved for exceptional circumstances where MRI is unavailable, contraindicated, or nondiagnostic, and the clinical suspicion remains high with significant concern for advanced or ruptured infection, acknowledging the associated fetal radiation risks [39].

The evidence also clarifies the role of diagnostic laparoscopy. In the era of advanced MRI, the rate of purely diagnostic laparoscopy (i.e., surgery without convincing preoperative imaging evidence) should plummet. The study by Lukenaite et al. [17] provides a clear before-and-after picture of this shift. However, laparoscopy retains a role when imaging is equivocal but clinical suspicion is compelling, or when an alternative surgical pathology (like adnexal torsion, as in the case reported by Mackay et al.) is suspected [39]. Furthermore, laparoscopy is the preferred therapeutic surgical approach for confirmed AA in the first two trimesters, associated with less postoperative pain, shorter hospitalization, and potentially a lower risk of thromboembolic events compared with open surgery [38]. The decision to operate must always involve a multidisciplinary team including obstetricians, surgeons, and radiologists to weigh the risks of surgical intervention against the risks of a missed or delayed diagnosis of AA, which can lead to perforation, peritonitis, preterm labor, and fetal loss [2].

Limitations

Given the currently available evidence, this systematic review has several limitations. First, selection bias and confounding are hazards introduced by the prevalence of retrospective research designs in the included studies. The QUADAS-2 assessment indicated low risk in the test interpretation domains. Second, the studies differ significantly in sample size, diagnostic procedures, MRI sequencing, and the specific cutoff values used for clinical ratings and laboratory markers. This heterogeneity precluded a formal meta-analysis to generate pooled statistical estimates of diagnostic accuracy. Third, the reference standard was not uniformly applied; while most studies used histopathology, some in the control groups relied on clinical follow-up, which could lead to misclassification if patients had self-limited appendicitis. Finally, many novel biomarkers and indices (e.g., SII and PGIndex) have been reported in single-center studies and require external validation across diverse populations before they can be widely adopted in clinical practice.

## Conclusions

Although diagnosing AA in pregnant women is still a challenging clinical problem, current research points to a straightforward and efficient diagnostic process. Clinical scoring systems derived from nonpregnant populations have limited PPV and should not be used in isolation. Basic laboratory markers are supportive but nondiagnostic. Ultrasonography remains a valuable first-line tool but is frequently inconclusive. Magnetic resonance imaging has emerged as the definitive noninvasive diagnostic test, characterized by high specificity and an exceptional NPV that can safely rule out appendicitis and drastically reduce the rate of unnecessary surgical interventions. Novel composite laboratory indices show promise for improving diagnostic precision and warrant further prospective validation. Large, prospective, multicenter studies should be the main focus of future research to create uniform MRI reporting standards, improve pregnancy-specific scoring systems, and verify diagnostic algorithms. Ultimately, the cornerstones of effective clinical management to guarantee the health of both the mother and the fetus are a high index of suspicion, prompt use of sequential imaging (US followed by MRI if necessary), and multidisciplinary consultation.
